# Lysophosphatidic Acid Improves Human Sperm Motility by Enhancing Glycolysis and Activating L-Type Calcium Channels

**DOI:** 10.3389/fendo.2022.896558

**Published:** 2022-07-12

**Authors:** Yinlam Li, Li Jin, Yanquan Li, Jianing Qian, Zhengquan Wang, Xiaoguo Zheng, Chong Xie, Xuelian Zhang, Hefeng Huang, Yuchuan Zhou

**Affiliations:** ^1^ International Peace Maternity and Child Health Hospital, School of Medicine, Shanghai Jiao Tong University, Shanghai, China; ^2^ Shanghai Key Laboratory of Embryo Original Diseases, Shanghai, China; ^3^ Obstetrics & Gynecology Hospital, Institute of Reproduction and Development, Fudan University, Shanghai, China; ^4^ State Key Laboratory of Genetic Engineering, School of Life Science, Fudan University, Shanghai, China

**Keywords:** lysophosphatidic acid, human spermatozoa, sperm motility, glycolysis, LPA receptors, calcium channels

## Abstract

Until now, the molecular mechanisms underlining sperm motility defect causing male infertility are still poorly understood. Safe and effective compounds or drugs that can improve sperm motility are also very limited. Lysophosphatidic acid (LPA) is a naturally occurring phospholipid and a bioactive intermediate with multiple biological activities. It has been detected in various body fluids such as serum, plasma, saliva, tears, blister fluids, hen egg white, and ascites from patients with ovarian cancer. LPA is also abundant in seminal plasma and follicular fluid. It enhances follicle stimulation, improves oocyte fertilization, and promotes early embryonic development and embryo implantation. However, the physiological role of LPA in the male reproductive system remains unknown. Here, our study showed that LPA significantly improved the motility parameters of human sperm hyperactivation in a dose-dependent manner. The LPA-induced elevation of sperm motility is dependent on bovine serum albumin (BSA) but independent of the classical BSA-induced sAC/cAMP/PKA signaling pathway. The enhancement of sperm motility by LPA could not be blocked by CCCP, a respiratory inhibitor suppressing mitochondrial ATP production. Moreover, LPA improved the activity of triosephosphate isomerase in glycolysis. Meanwhile, LPA treatment significantly increased ATP and phosphoenolpyruvate levels and decreased ADP content during sperm glycolysis. Notably, none of known or identified LPA receptors was detected in human sperm. Further investigations showed that LPA promoted sperm motility through L-type calcium channels. In summary, this study revealed the involvement of LPA in the regulation for human sperm motility by enhancing glycolysis and activating L-type calcium channels. The current findings may shed new light on the understanding of causes of asthenozoospermia, and indicate that LPA could be used as a novel therapeutic agent to improve sperm function and fertilizing capacity.

## Introduction

Globally, approximately 15–20% of childbearing-age couples are infertile, and male factors contribute to half of all infertility cases (1–3). Male infertility imposes enormous social, psychological, and financial pressure on married couples (4, 5). Low sperm count, abnormal morphology, and poor sperm motility contribute to male fertilization defects. Approximately 20% of male infertility cases are caused by asthenozoospermia, which is mainly manifested by reduced sperm motility (6–9).

Currently, there are no drugs that can effectively treat or cure male infertility, and assisted reproductive technology (ART) including intrauterine insemination (IUI), *in vitro* fertilization (IVF), and intracytoplasmic sperm injection (ICSI) is the only available option. Low sperm motility is not only unconducive to natural conception, but also leads to ART failure. Enhancement of sperm motility improves IUI and IVF success rates (10) and reduces dependence on ICSI, which is associated with greater expenditures and a higher risk of birth defects (11, 12). In addition, improving sperm motility is helpful for rapid selection of viable spermatozoa in the ICSI procedure for patients with asthenospermia or frozen-thawed sperm with poor or no motility (13). Therefore, it is very important to enhance sperm motility to improve male fertilizing capacity and ART success rate.

Researchers are trying to identify compounds or pharmacological drugs to improve the motility capability of fresh and frozen human sperm (14–16). Several studies have revealed that diverse endocrine and physiological stimuli such as progesterone (17), follicular fluid (18), and cumulus cells (19) increase the motility of spermatozoa *in vitro*. Currently, the most common stimulants used for human sperm motility are caffeine and pentoxifylline (PTX), two non-specific inhibitors of phosphodiesterases (20, 21). However, studies have indicated that PTX shows low specificity, induces a precocious acrosomal response, and has detrimental effects on embryonic development, limiting its clinical use as a sperm motility-enhancing agent (22–24). Subsequently, some reports indicated that biotin can enhance sperm motility to improve the fertilization potential and preimplantation embryo development and can be considered as a safe alternative to PTX (22, 25). Thus, the discovery of more novel molecules that enhance sperm motility without any adverse effects on oocyte or embryo development will have potential therapeutic value in treating infertility (26).

Lysophosphatidic acid (LPA) is a natural and intercellular bioactive glycerophospholipid with diverse biological activities. It is widely found in many different body fluids, such as blood, follicular fluid, tears, seminal plasma, and saliva and ascites from patients with ovarian cancer (27, 28). In the past few decades, studies have found that LPA induces a variety of physiological processes such as cell proliferation, platelet aggregation, stimulation of DNA synthesis, apoptosis prevention, cell migration, cytokine and chemokine secretion and neurite retraction, morphological change in the cytoskeleton, and evocation of an inward Cl^-^ current in *Xenopus* oocytes (29–31). Furthermore, elevated LPA has been shown to be related to pathologies such as breast, prostate, and pancreatic cancers (32, 33) and neuropathic pain (34). The actions of LPA are now thought to be mediated through six cognate G-protein-coupled receptors. LPA receptors also play a vital role in cancer, neurological disease, cardiovascular disease, liver disease, and metabolic disease (35). In the reproductive system, studies in humans and different species of animals have identified that LPA promotes follicle stimulation, improves oocyte fertilization, and promotes early embryonic development and embryo implantation (27, 36, 37). LPA stimulates egg maturation *in vitro* and ovum transport in an *ex vivo* system (38, 39). However, the physiological and pathological roles of LPA in the male reproductive system remain poorly understood. Some reports have shown that LPA content is high in seminal plasma (27, 28, 30). Another study in mice found that the LPA receptors, specifically LPA1, LPA2, and LPA3, were significantly expressed in the testes (40). In addition, *Lpa1-3* triple knockout mice showed age-dependent sperm production loss, increased apoptosis, and decreased germ cell proliferation (40). These reports suggest that LPA and its receptors exert their important functions in the male reproductive system. In this study, we investigate the role of LPA in sperm motility and attempt to elucidate the molecular mechanisms by which it regulates sperm motility.

## Materials and Methods

### Chemicals/Reagents

Biggers, Whitten, and Whittingham (BWW) medium for human sperm collection and culture was prepared according to the fifth edition of the WHO Laboratory manual for the examination and processing of human semen and our previously published report (41). All saline components used in BWW medium and LPA (L7260) were purchased from Sigma-Aldrich (MO, USA). Anti-phosphotyrosine monoclonal antibody 4G10, anti-α-tubulin monoclonal antibody, and goat anti-mouse IgG–H&L chain-specific peroxidase conjugate were purchased from Merck Millipore Corporation (Billerica, MA, USA). Anti-p-PKA substrate antibody was purchased from Cell Signaling Technology Corporation (Danvers, MA, USA). Nifedipine, Verapamil, mibefradil, NNC 55-0369, β-cyclodextrin, and bovine serum albumin (BSA) were obtained from Sigma-Aldrich. JC-1 was from Molecular Probe (Eugene, OR). Anti-LPA1 antibody (ab166903), anti-LPA2 antibody (ab135980), anti-LPA3 antibody (ab23692), anti-LPA4 antibody (ab183076) and anti-LPA5 antibody (ab77680) were purchased from Abcam (MA, USA). Anti-LPA6 antibody (orb30731) was purchased from Biorbyt (UK).

### Ethical Approval

The collection of semen samples and all experiments in this study were approved by the Ethics Committee on human subjects of International Peace Maternity and Child Health Hospital (GKLW2018-03).

### Sperm Sample Collection and Treatments

Seminal fluid was obtained by masturbation into a sterile plastic container after 3–5 days of sexual abstinence from participants who visited the International Peace Maternity and Child Health Hospital, Shanghai, China, for diagnostic semen analysis between January 2019 and January 2021. Normozoospermic (sperm concentration ≥15 million cells/mL, progressive motility ≥32%, total motility ≥40%) and asthenozoospermic (sperm concentration ≥15 million cells/mL, progressive motility <32%, total motility <40%) men were classified according to the World Health Organization (WHO) reference intervals that are based on manual assessments. Samples were allowed to liquefy at room temperature for at least 0.5 h. Following diagnostic semen analysis, surplus samples were allocated for research. The spermatozoa were directly washed with BWW solution (94.8 mM NaCl; 4.8 mM KCl; 1.7 mM CaCl_2_, 1.2 mM MgSO_4_, 1.2 mM KH_2_PO_4_, 5.5 mM glucose, 13.21 mM sodium lactate, 0.27 mM sodium pyruvate, 25 mM NaHCO_3_, and 3.5 mg/mL BSA), centrifuged, collected, and purified. Then, the spermatozoa were re-suspended in BWW medium and adjusted to a final concentration of 10–20 × 10^6^ cells/mL. According to the needs and designs of the different tests, the sperm suspension was divided into equal parts and treated by LPA or inhibitors or both LPA and inhibitors, and then incubated at 37°C in a 5% CO_2_ incubator for specific times. In some experiments, glucose-free media were used, and glucose was added back to final concentrations if necessary. In some experiments, BSA-free medium was used, or β-cyclodextrin was used to replace BSA in capacitating media. To verify the uncoupling effect of CCCP on mitochondria, sperm were treated by mitochondrial uncoupler CCCP (10 μM) for one hour and stained with JC-1 (5 μM). After washing, the fluorescence of sperm was observed and assessed under the microscope.

### Assessment of Sperm Motility

Sperm concentration and motility were assessed using computer-assisted sperm analysis (CASA; Hamilton-Thorne, Beverly, MA, USA). After the treatment with LPA or inhibitors or both LPA and inhibitors, an aliquot of 10 μl of each sperm sample was transferred to a sperm-counting chamber and sperm motility parameters were recorded. A minimum of 200 sperm from 10 randomly selected fields were assessed in each chamber. The playback function of the system was used to check its accuracy.

### Animal Samples

C57 mice aged 8-12 weeks were sacrificed by cervical dislocation, and then the heart, spleen, and testis were isolated and immediately frozen in liquid nitrogen. The caudal epididymis was dissected and placed in BWW medium at 37 °C to release sperm. After washing and centrifugation, sperm were frozen in liquid nitrogen and stored at -80 °C until use.

### Western Blot Analysis

Tissue and sperm protein expression was analyzed by immunoblotting as previously described (41). In brief, total protein extracts obtained from the tissues or spermatozoa were separated by electrophoresis on 12% (w/v) sodium dodecyl sulfate-polyacrylamide gels, transferred onto polyvinylidene fluoride membranes, and probed with primary antibodies as follows: anti-LPA1–6 (1:1000 dilution); anti-phosphotyrosine (1:10000 dilution); anti-phospho-PKA substrates (1:1000 dilution) and anti-α-tubulin (1:10000 dilution). The bound IgG was detected with goat-anti-rabbit (Sigma) or goat-anti-mouse (Millipore) antibody conjugated to horseradish peroxidase at a dilution of 1:10000. ECL Plus (Amersham) were used for chemiluminescence detection.

### Determination of Energy Metabolomics

Sperm were treated with 100 µM LPA and collected, and then metabolites were extracted for energy metabolism detection as described in a previous report (42). In brief, 1 mL of pre-cooled methanol/acetonitrile/water (2:2:1, V/V/V) and 10 µL of internal standard were added to the samples, respectively, and ultrasound was performed in an ice bath for 20 min. The homogenate was then centrifuged at 14000 g at 4 °C for 20 min, and the supernatant was removed and dried in a vacuum centrifuge. These samples were collected and sent for metabolon-associated energy metabolism analysis (Applied Protein Technology, Shanghai, China). The samples were separated by ultra-high-performance liquid chromatography (UHPLC, Agilent 1290 Infinity II LC System), and a quality control sample was set for each interval of a certain number of experimental samples in the queue to test and evaluate the stability and repeatability of the system. The control of the sample cohort was set with a standard mixture of glycometabolites for correction of chromatographic retention time. This was followed by mass spectrometry using a 5500 QTRAP mass spectrometer (AB SCIEX) in negative ion mode. Finally, the peak area and retention time were extracted with Multiquant software, and metabolites were identified after correction with standard.

### Determination of Enzyme Activity

After 100 µM LPA treatment, the sperm samples were centrifuged to remove the supernatant and the pellet was re-suspended with 200 µL phosphate-buffered saline precooling on ice for sonication. Briefly, the cold sperm suspension was sonicated by three 30-sec pulses and referred to as sperm sonicate. The samples were then centrifuged at 13000 rpm at 4 °C for 20 min to collect the supernatant. The triosephosphate isomerase (TPI) assay was performed on a microplate reader at OD=450 nm using a kit (Abcam, Cambridge, USA; cat no: ab197001) according to the manufacturer’s instructions and the report as described previously (43). The activity of glycolytic enzymes glyceraldehyde-3-phosphate dehydrogenase (GAPDH) in supernatant were measured according to the manufacturer’s instructions (Solarbio, Beijing, China). The ELISA Analyzer (BioTek, USA) was preheated to 37°C and the wavelength was adjusted to 340 nm. The samples were fully mixed with the working solution provided by the GAPDH detection kits (Solarbio, Beijing, China) in the 96-well plate. At 340 nm wavelength, the absorbance values A1 and A2 of the sample mixture before and after incubation at 37 °C for 5 minutes were measured respectively. Finally, the activity of enzyme was calculated.

### Statistical Analysis

Data are shown as mean ± standard error of the mean (SEM), with *n* referring to the number of independent experiments performed using sperm samples from at least three different donors. Statistical analysis was performed using GraphPad Prism 8 (Prism, La Jolla, USA). Statistical significance of the difference between control and LPA-treated conditions was evaluated using one-way ANOVA when comparing each of the different doses with a single control, respectively. A paired t-test was used to analyze the data between control and single-dose treatment groups. *P <*0.05 was considered to indicate statistical significance.

## Results

### Lysophosphatidic Acid Increases Sperm Motility

**Figure 1:**
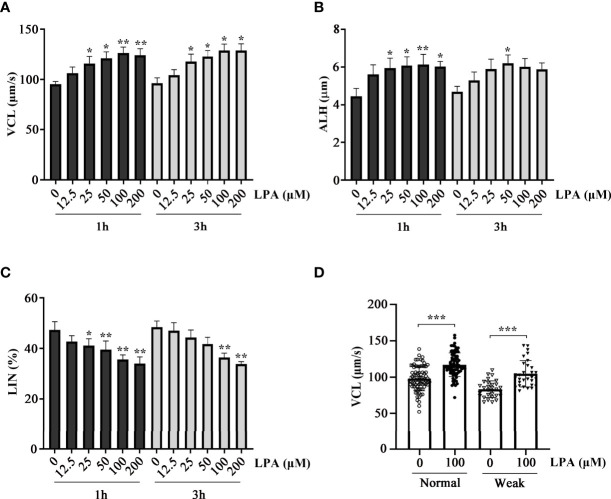
Effect of lysophosphatidic acid (LPA) on human sperm motility. **(A–C)** Dose-dependent effects of LPA on sperm motility. Normal human spermatozoa were treated with LPA (0, 12.5, 25, 50, 100, and 200 µM) for 1 h and 3 h in the Biggers, Whitten, and Whittingham (BWW) medium. Parameters of sperm motility, VCL **(A)**, ALH **(B)**, and LIN **(C)**, were examined using computer-assisted sperm analysis. Results are expressed as mean ± SEM (*n* = 6). *p < 0.05, **p < 0.01 as compared with the corresponding control (0 µM); VCL: curvilinear velocity; ALH: amplitude of lateral head displacement; LIN: linearity. **(D)** Effect of LPA (100 µM) on the VCL of spermatozoa from healthy individuals (*n* = 80) and patients with asthenozoospermia (*n* = 30). The motility was assessed after spermatozoa were treated with LPA (0 and 100 µM) for 1 h in the BWW medium. Results are expressed as mean ± SEM. ***p < 0.001 as compared with the corresponding control (0 µM).

To test the effect of LPA on sperm function *in vitro*, we used five different concentrations of LPA (0, 12.5, 25, 50, 100, and 200 µM) to study its effect on normal sperm selected according to WHO standards. Sperm hyperactive motility related parameters such as curvilinear velocity (VCL), linear velocity (LIN), and amplitude of lateral head displacement (ALH) were measured after incubation with LPA for 1 h and 3 h, respectively. The results showed that LPA significantly increased VCL (**Figure 1A**) and ALH (**Figure 1B**) and reduced LIN (**Figure 1C**) in a dose-dependent manner, and the changes of motility parameters were the highest when samples were incubated with 100 µM LPA. Furthermore, LPA at the dose of 100 µM evidently elevated the VCL of sperm from normozoospermia and asthenozoospermia (**Figure 1D**). Unless otherwise noted, we selected the VCL as a measurement parameter and used the dosage of 100 µM LPA and the incubation time of 60 minutes for the subsequent experiments in this study.

### Lysophosphatidic Acid-Induced Increase in Sperm Motility Is Dependent on Bovine Serum Albumin but Independent of the sAC/cAMP/PKA Signaling Pathway

**Figure 2:**
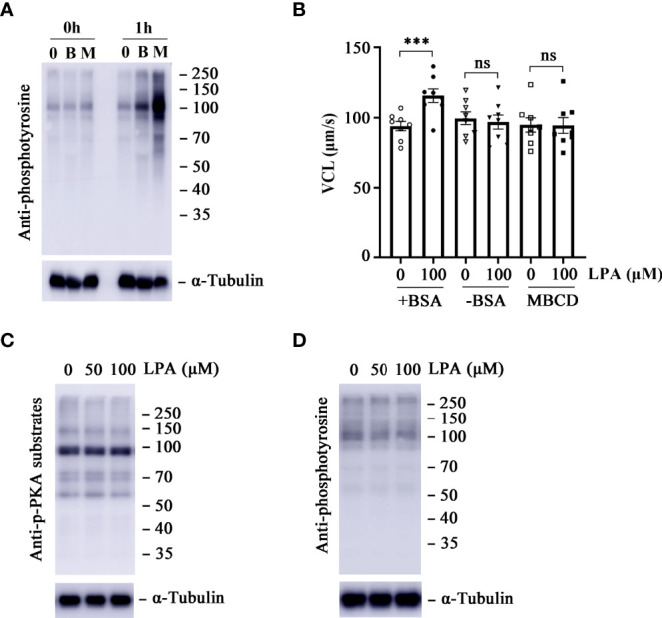
Lysophosphatidic acid (LPA)-induced increase in sperm motility is dependent on bovine serum albumin (BSA) but independent of the protein kinase A (PKA) signaling pathway. **(A)** Effects of BSA (3.0 mg/mL) and β-cyclodextrin (3.0 mg/mL) on sperm capacitation-associated protein tyrosine phosphorylation. Spermatozoa were incubated in capacitating medium and collected for western blot at 0 h and 1 h α-tubulin served as the loading control. The western blot is representative of three independent experiments. B: BSA, M: β-cyclodextrin. **(B)** The curvilinear velocity (VCL) of spermatozoa was determined after they were treated with 100 µM LPA for 1 h in the absence or presence of BSA (3.0 mg/mL) and β-cyclodextrin (3.0 mg/mL) in the medium. Results are expressed as mean ± SEM (*n* = 8). ***p < 0.001 as compared with the control; ns: no significance. MBCD: β-cyclodextrin. **(C, D)** Measurement of PKA substrate phosphorylation **(C)** and capacitation-associated protein tyrosine phosphorylation **(D)** of spermatozoa. Spermatozoa were treated with LPA (0, 50, and 100 µM) for 1 h in the Biggers, Whitten, and Whittingham (BWW) medium. α-tubulin served as the loading control. The western blot is representative of three independent experiments.

It has been demonstrated that the activity of LPA depends on albumin and other LPA-binding proteins in biological fluids (30). To test whether stimulation of sperm motility by LPA is associated with BSA, sperm VCL parameters were measured after treating the sperm with 0 and 100 µM LPA in the presence or absence of BSA in the medium at 37 °C for 1 h. LPA significantly increased the VCL in the presence of BSA but not in the absence of BSA (**Figure 2B**). BSA or β-cyclodextrin (β-CD) in sperm culture medium has been used as a sink to remove cholesterol from the plasma membrane and subsequently to promote sperm membrane fluidity (44). Then, the entry of bicarbonate and calcium from the medium into the sperm cells activates soluble adenylyl cyclase (sAC) and leads to an increase in cyclic adenosine 3′,5′-monophosphate (cAMP) levels, subsequent activation of cAMP-dependent protein kinase A (PKA), and protein tyrosine phosphorylation. Our control WB experiments showed the increased protein tyrosine phosphorylation level of sperm treated by β-cyclodextrin or BSA (**Figure 2A**). We attributed this to activation of the sAC/PKA/cAMP pathway, which results in increased beat frequency or hyperactivity of spermatozoa (45). To determine whether the LPA-induced effects on sperm VCL are dependent on the sAC/cAMP/PKA signaling pathway, we measured the change in tyrosine phosphorylation and PKA substrate phosphorylation in sperm treated with 50 and 100 µM LPA. There were no significant differences in PKA substrate phosphorylation (**Figure 2C**) and protein tyrosine phosphorylation (**Figure 2D**) between the experimental and control groups. Furthermore, the experiment in which BSA was replaced with β-cyclodextrin showed that LPA had no positive effect on sperm motility parameters in the absence of BSA (**Figure 2B**).

### Lysophosphatidic Acid-Induced Increase in Sperm Motility Is Dependent on the Presence of Glucose in the Medium

**Figure 3:**
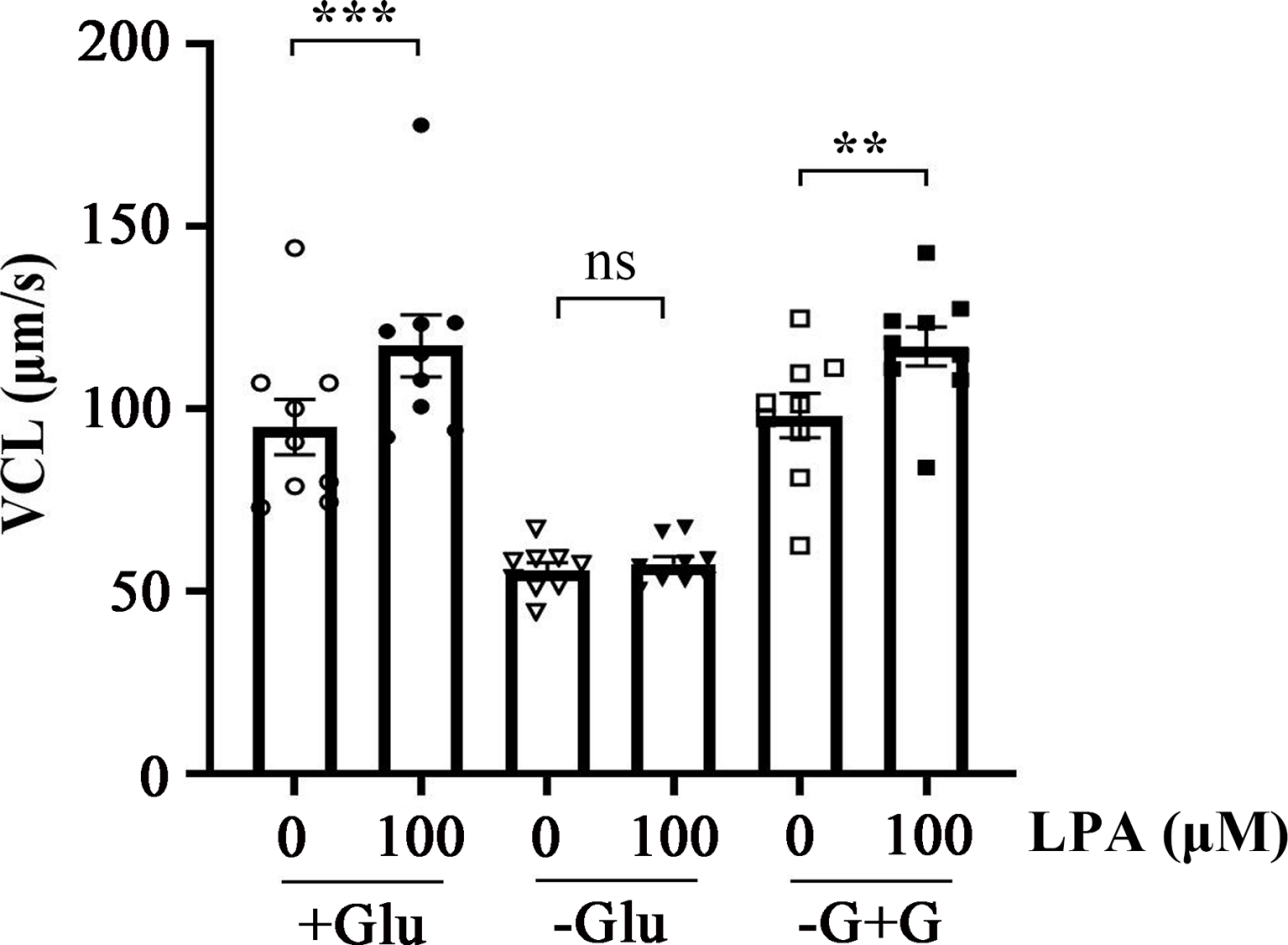
Lysophosphatidic acid (LPA)-induced increase in sperm motility is dependent on glucose in the medium. Spermatozoa were treated with 100 µM LPA for 1 h in the absence or presence of glucose in the medium. Results are expressed as mean ± SEM (*n* = 9). **p < 0.01, ***p < 0.001 as compared with the corresponding control (0 µM). Glu: glucose; -G+G: Glucose was added to the medium without glucose for 15 minutes.

Energy metabolism plays a key role in maintaining sperm motility. The ATP required for sperm movement is derived from glycolysis and mitochondrial respiration (46). Glucose is an important factor for human sperm fertilizing capacity (47). To determine whether the stimulation of sperm motility by LPA is dependent on glucose, we examined the effect of 100 µM LPA on VCL in the absence and presence of glucose in the medium at 37 °C for 1 h. **Figure 3** displays that LPA significantly increased sperm VCL in the presence of glucose in the medium. When the sperm were incubated in the absence of glucose for 1 h, the acceleration effect of LPA on VCL disappeared. Whereas glucose was added to the glucose-free medium for 15 minutes, LPA significantly enhanced sperm motility (**Figure 3**).

### Lysophosphatidic Acid Increases Sperm Motility by Enhancing Glycolysis

**Figure 4:**
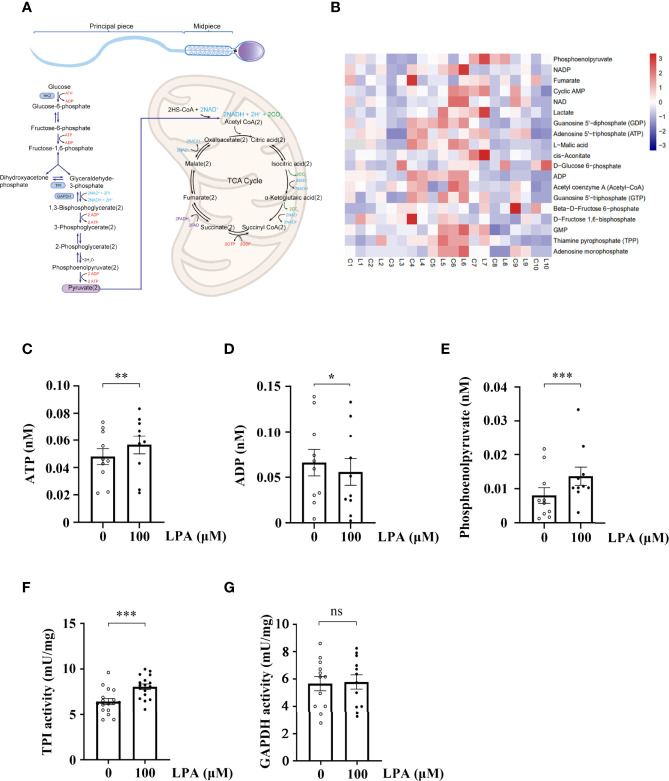
Lysophosphatidic acid (LPA) accelerates sperm glycolysis. **(A)** Diagram depicting energy production in sperm. **(B)** Heatmap of the metabolite status in sperm treated by LPA. The sperm sample from a single person were divided into two equal parts, and spermatozoa were treated with LPA (0 and 100 µM) for 1 h in the Biggers, Whitten, and Whittingham (BWW) medium and collected for targeted glycometabolomics (*n* = 10). C: control, L: LPA (100 µM). The x-axis represents the comparative change of each sperm sample before (C1-10) and after (L1-10) LPA treatment. **(C–E)** The levels of ATP **(C)**, ADP **(D)**, and phosphoenolpyruvate **(E)** in spermatozoa treated with LPA (0 and 100 µM) for 1 h in BWW medium. Data are presented as mean ± SEM. *n* = 10. *p < 0.05 and **p < 0.01, ***p < 0.001 compared with the corresponding control (0 µM). **(F)** Change in triosephosphate isomerase (TPI) activity in sperm before and after LPA treatment. Data are presented as mean ± SEM. *n* = 17. ***p < 0.001 compared with the corresponding control (0 µM). **(G)** Change of glyceraldehyde-3-phosphate dehydrogenase (GAPDH) activity in sperm before and after LPA treatment. Data are presented as mean ± SEM. *n* = 12. ns: there was no statistical significance compared with the corresponding control (0 µM).

**Figure 5:**
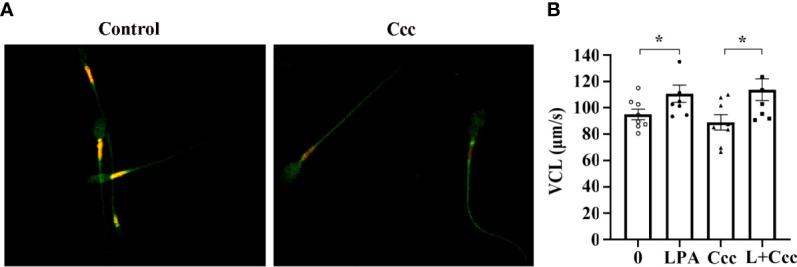
Effect of mitochondrial uncoupling on LPA-stimulating sperm motility. **(A)** The activity of mitochondria uncoupled by 10 µM CCCP (Ccc) was measured by 5 µM JC-1. Microscopic analysis showed the change of red/orange fluorescence of sperm treated with CCCP. **(B)** Spermatozoa were treated with 100 µM LPA (L) for 1 h in the absence or presence of 10 µM CCCP (Ccc), an uncoupler used to inhibit mitochondrial ATP production. The curvilinear velocity (VCL) of sperm motility was assessed by computer-assisted sperm analysis. Data are presented as mean ± SEM. *n* = 8. *p < 0.05 compared with the corresponding control (0 µM).

Given that the promoting effect of LPA on sperm motility is dependent on glucose (**Figure 3** and **4A**), we performed a targeted glycometabolome analysis. As shown in **Figure 4B**, nineteen glucose metabolites were detected, and the change status of each metabolite in sperm before (C1-10) and after (L1-10) LPA treatment were displayed. Among them, the levels of ATP and phosphoenolpyruvate was significantly increased (**Figure 4C, E**), when the level of ADP was markedly decreased in the LPA-treated group (**Figure 4D**). Other metabolites did not alter significantly between the experimental and control groups (data not shown). Determination of enzyme activity showed that LPA significantly promoted the activity of triosephosphate isomerase (TPI) (**Figure 4F**) but had no effect on glyceraldehyde-3-phosphate dehydrogenase activity (**Figure 4G**). Furthermore, CCCP, a respiratory inhibitor, was used to test the effect of mitochondrial uncoupling on LPA-stimulating sperm motility. As shown in **Figure5**, Mitochondrial respiratory chain can be effectively uncoupled by CCCP (**Figure 5A**). However, sperm motility stimulated by LPA could not be significantly blocked by CCCP (**Figure 5B**).

### Expression of Lysophosphatidic Acid Receptors in Sperm

**Figure 6:**
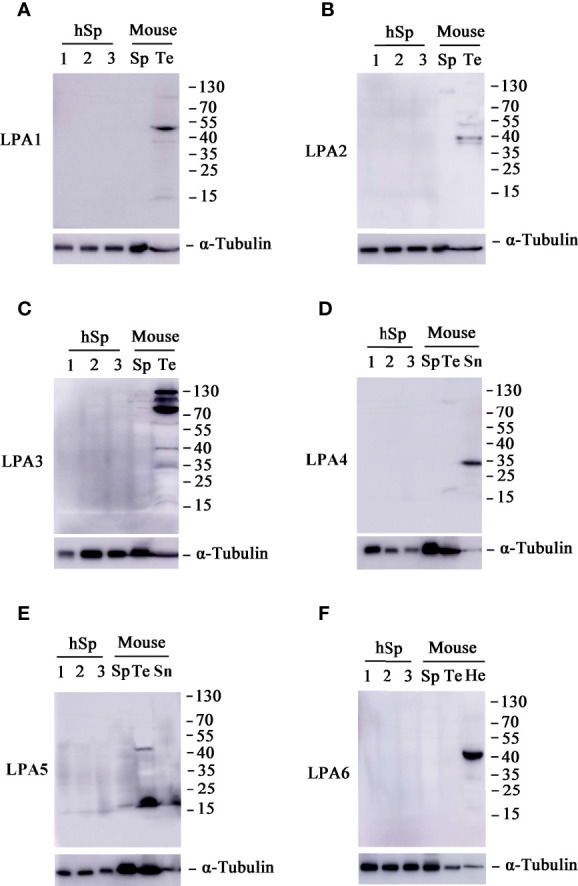
Expression patterns of lysophosphatidic acid (LPA) receptors in sperm. Western blot analyses of proteins LPA1 **(A)**, LPA2 **(B)**, LPA3 **(C)**, LPA4 **(D)**, LPA5 **(E)**, and LPA6 **(F)** in human sperm (hSp), mouse sperm (Sp) and testis tissue (Te). Spleen (Sn) tissues were also included in the experiments for LPA4 and LPA5, whereas heart **(He)** tissues for LPA6. The blot was probed with monoclonal antibodies against α-tubulin to assess protein loading.

Previous research suggests that the diverse and numerous physiological effects of LPA are driven by extracellular signaling through at least six 7-transmembrane G protein-coupled receptors (GPCRs). These receptors, named LPA1–6, are coupled with at least one or more of the four Gα proteins (G12/13, Gq/11, Gi/o, and Gs) that initiate various signaling cascades (48). It has previously been shown that LPA1, LPA2, and LPA3 are highly expressed in the testis, whereas LPA4 and LPA5 expression is low (40). Therefore, we investigated the protein expression level of LPA receptors in human and mouse sperm. As shown in **Figure 6**, none of the six LPA receptors was found in human and mouse sperm.

### Lysophosphatidic Acid Promotes Sperm Motility Through L-Type Calcium Channels

**Figure 7:**
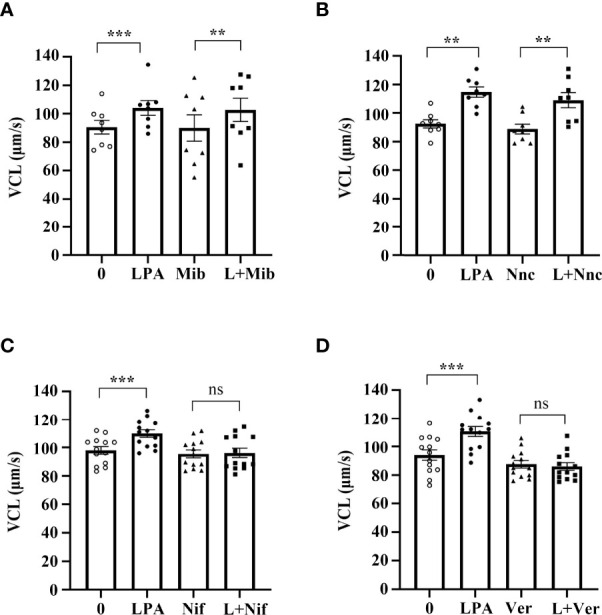
Lysophosphatidic acid (LPA) improves sperm motility *via* L-type calcium channels. **(A, B)** Spermatozoa were treated with 100 µM LPA for 1 h in the absence or presence of T-type calcium channel inhibitor mibefradil (Mib; 30 µM) **(A)** or NNC55-0369 (Nnc; 10 µM) **(B)**. Curvilinear velocity (VCL) was then assessed by computer-assisted sperm analysis. Data are presented as mean ± SEM (*n* = 8). **p < 0.01, ***p < 0.001 compared with the corresponding control (0 µM). **(C, D)** Spermatozoa were treated with 100 µM LPA for 1 h in the absence or presence of L-type calcium channel inhibitor nifedipine (Nif; 200 µM) **(C)** or verapamil (Ver; 200 µM) **(D)**. Curvilinear velocity (VCL) was then assessed by computer-assisted sperm analysis. Data are presented as mean ± SEM (*n* = 13). ns: no statistical significance, ***p < 0.001 compared with the corresponding control (0 µM).

Ca^2+^ and calcium channels control the flagellum beat and swimming behavior of sperm and are essential for sperm motility (49). To investigate whether LPA-induced increase in VCL is associated with Ca^2+^ channels, we used the inhibitors of different Ca^2+^ channels to block LPA activity. The results demonstrated that mibefradil (**Figure 7A**) and NNC 55-0396 (**Figure 7B**), inhibitors of T-type calcium channels, could not abolish the stimulating effect of LPA on sperm VCL. However, in the presence of nifedipine and verapamil, inhibitors of L-type calcium channels, LPA lost the ability to promote sperm motility (**Figure 7C, D**).

## Discussion

Ejaculated sperm are still functionally immature and must undergo capacitation in the female reproductive tract or in chemically defined media to acquire the ability to fertilize eggs. During capacitation, sperm acquire hyperactivated motility, and the sperm movement pattern changes from symmetric and low-amplitude movement to asymmetric and large-amplitude movement. The hyperactivated motility may allow sperm to swim faster and generate sufficient force to penetrate the cumulus cells and zona pellucida during fertilization (50). Previous reports have shown that seminal fluid and follicular fluid contain a considerable quantity of LPA (31) (28). In the present study, the results showed that LPA improved the VCL and ALH parameters of sperm, suggesting that LPA is a potential stimulus during sperm-egg interaction.

Asthenozoospermia is one of the major causes of male infertility, and high motility is a reliable predictor of success rate for the fertilization potential of spermatozoa *in vivo* and in ART (51, 52). Therefore, the search for agents that enhance sperm motility has important clinical implications. Previously, attempts have been made to increase sperm motility by using endogenous molecules such as progesterone, follicular fluid, and cumulus cells (17–19, 22). In the present investigation, we observed that LPA enhanced the motility of human spermatozoa *in vitro*, which indicates the potential application of LPA as human sperm motility enhancer. In addition, we found no LPA receptors on human sperm, indicating that LPA stimulates sperm motility *via* a mechanism independent of its receptor. The present results showed that sperm can be used as a good model to study the receptor-independent molecular mechanism of LPA actions.

It is widely accepted that sperm motility is closely associated with the activation of the sAC/cAMP/PKA signaling pathway (45, 53). Stimulation of cAMP production increases the percentage of motile sperm and improve the motility of frozen-thawed human sperm (16, 54). BSA or β-cyclodextrin (β-CD) in sperm culture medium initiates this signaling pathway and activates sAC, resulting in increased cAMP level, subsequent PKA activation, and protein tyrosine phosphorylation. Then, the sperm enter the stage of capacitation or hyperactivation (44, 50). Previous reports have shown that serum albumin and other LPA-binding proteins can modulate the cellular function of LPA (30, 55). Another study indicates that albumin-bound LPA in human serum activates membrane currents in *Xenopus* oocytes and neurite retraction in PC12 cells (56). Our present results demonstrated that LPA-induced increase in sperm motility depended on the presence of BSA in the culture medium. However, tyrosine phosphorylation and PKA substrate phosphorylation in the sAC/cAMP/PKA signaling pathway were not altered with LPA-induced increase in sperm motility. By replacing BSA with β-cyclodextrin, we excluded the possibility that the effect of LPA-dependent BSA was due to the change in sperm membrane fluidity. The findings suggest that the mechanism of action of LPA differs from that of already characterized compounds known to stimulated sperm motility, such as pentoxifylline or LY294002, which act mainly on the kinase pathway ending with PKA or by inhibiting sperm phosphodiesterases to increase the cAMP concentration within the cell (57). Thus, LPA stimulates sperm motility independent of the classical sAC/cAMP/PKA signaling pathway.

Sperm motility depends on the energy availability in the metabolic pathway of oxidative phosphorylation in the mitochondria or glycolysis or both (53). Human sperm motility is not only associated with mitochondrial functional status but also depends, at least partly, on glycolysis. Previous evidence suggests that an increase in human sperm motility requires a corresponding increase in mitochondrial activity (58, 59). A blockade of the mitochondrial electron transport chain and ATP synthase by specific inhibitors significantly decreases human sperm motility (60). When human sperm are deprived of glucose or when glycolysis is blocked, sperm show significantly reduced motility (60–62). In the present study, we found that LPA promotes sperm motility *via* mechanism dependent on glucose in the culture medium. Furthermore, we used targeted energy metabolomics to investigate the effect of LPA on glucose metabolism and found no significant changes in glucose metabolites in the oxidative phosphorylation pathway. However, LPA significantly promoted the production of glycolytic products and enzyme activity of glycolysis. The action of mitochondrial uncoupling CCCP also ruled out the effect of LPA on sperm motility by regulating oxidative phosphorylation pathway in mitochondria. These results suggest that LPA enhances sperm motility mainly by activating the glycolytic pathway. Notably, LPA significantly induces the acrosome reaction in bovine sperm, but it does not affect sperm motility (63). This finding is indeed consistent with our results. In bovine sperm, oxidative phosphorylation appears to be the only source of ATP for motility (64). Therefore, these observations validate our conclusion that LPA acts on glycolysis to enhance sperm motility.

A previous report showed that LPA promotes survival of T lymphoma cells by altering the expression of glucose metabolism regulatory molecules, including glucose transporter 3, hexokinase II, pyruvate kinase muscle isozyme 2, monocarboxylate transporter 1, and pyruvate dehydrogenase kinase 1 (65). Because sperm chromosomes are highly condensed, and transcription and translation have basically stopped (66), we decided to assess the activity of certain enzymes involved in glucose metabolism. The results showed that LPA significantly enhanced the activity of triosephosphate isomerase, a glycolysis-related enzyme, which was consistent with the LPA-induced decrease in ADP and increase in ATP and phosphoenolpyruvate in the glycolytic pathway. These results imply that LPA not only regulates the expression of genes related to glucose metabolism but also changes the activity of enzymes involved in glycolysis. Triose phosphate isomerase (TPI) is a reversible enzyme converting dihydroxyacetone phosphate into glyceraledehyde-3-phosphate. Previous reports indicated that TPI inhibitor decreased rat sperm motility by 50%, consistent with the hypothesis that the conversion of dihydroxyacetone phosphate into glyceraledehyde-3-phosphate increases the efficiency of the glycolytic process (67, 68). This suggests that TPI enzyme activity can reflect the activation of glycolytic pathway and regulate sperm motility. In our study, when LPA significantly increased the ATP level and motility of sperm, TPI activity was also markedly enhanced. We speculate that TPI enzyme activity is one of the manifestations of LPA activated glycolysis. However, we’re really not sure the exact direction of TPI action, and also cannot clarify the extent to which the increased TPI activity contributes to LPA-induced glycolytic ATP production and sperm motility. Therefore, further analysis will require an accurate determination of all intermediates associated with these steps, especially dihydroxyacetone phosphate and glyceraledehyde-3-phosphate. Thus, it is important to investigate and clarify which steps LPA acts on glycolysis in the future research.

LPA signaling is thought to be mediated through at least six isoforms of G-protein-coupled receptors, namely LPA1-6. To date, the distribution and expression of LPA receptors in sperm has remained unknown. It was reported that LPA1, LPA2, and LPA3 show high mRNA expression in mouse testis (40), whereas LPA4 was detected in human testis (69). In the present study, we found that LPA1, LPA2, LPA3, and LPA5 proteins were expressed in mouse testis, but none of the LPA receptors was detected in human and mouse sperm. A previous study demonstrated that sperm motility was not altered in *Lpa1-3* triple knockout mice (40). Thus, the lack of LPA receptors did not completely impair the fertilizing capacity of mice, suggesting that LPA action is associated with other signaling pathways or some unknown LPA receptors.

Recently, more and more studies have begun to unveil that LPA can directly or indirectly regulate the function and expression of ion channels such as N- and T-type Ca^2+^ channels, M-type K^+^ and Kir 2.1 K^+^ channels, TRPM2, TRPV1, TRPA1 channels as well as TREK-1, TREK-2, TRESK and TRAAK channels (70). It has also been demonstrated that T-type Ca^2+^ channels (Cav 3.1-Cav 3.3) are regulated by LPA and that such regulation may play a role in neuropathic pain (71). LPA triggers a rise in cytosolic calcium concentrations by opening a calcium channel in the erythrocyte plasma membrane (72). TMEM16F, a Ca^2+^-gated ion channel, is able to modulate LPA-induced calcium uptake and exposure of phosphatidylserine at the cell surface (73). In sperm, Ca^2+^ can activate sAC/cAMP/PKA pathway to promote protein tyrosine phosphorylation, thus accelerating sperm movement (74). In addition, cation Ca^2+^ channel, CatSper calcium channel, can regulate sperm motility by mediating calcium influx (75). In our present study, we excluded the role of sAC/cAMP/PKA pathway and CatSper in LPA action by examining the protein tyrosine phosphorylation and using mibefradil and NNC 55-0396, inhibitors of the sperm-specific CatSper channel. Then we further revealed by using L-type Ca^2+^ channel inhibitors that LPA-induced the increase of human sperm motility depends on the activation of L-type calcium channels. Thus, L-type calcium channels may be the key mediator of LPA action on sperm lacking LPA receptors.

Given that LPA can enhance glycolysis in our study, we speculate that there might be a dialogue mechanism between L-type calcium channels and glycolysis. Previous report showed that L-type calcium channels is necessary for support of synaptic function by lactate utilization in glycolysis (76). On the other hand, glycolytic ATP has been shown to preferentially regulate cardiac, skeletal and sarcoplasmic reticular Ca^2+^ transport (77). Furthermore, some reports have shown that glycolytic enzymes are colocalized with skeletal Ca^2+^ channels (78). Rabbit cardiac L-type calcium channel is preferentially regulated by ATP derived from glycolysis as opposed to oxidative phosphorylation (79). In normal prostate cells, verapamil, an L-type calcium channel blocker, diminishes glucose and glycolytic intermediate levels leading to ATP depletion. In contrast, in COLO 205 cells it enhances aerobic glycolysis and maintains ATP (80). Nifedipine, another L-type calcium channel blocker, enhanced glycolytic capacity in chondrocytes (81). These reports indicated that there was a cross-talk mechanism between L-type calcium channels and glycolysis. Therefore, it is reasonable to speculate that LPA regulates sperm movement through L-type calcium channels and its dialogue with glycolysis. Unfortunately, the relationship between L-type calcium channels and glycolysis in sperm is still unclear. At present study, we also have no sufficient experimental evidence to clarify this relationship. Therefore, it is necessary for future research to investigate which L-type calcium channels LPA acts on and how L-type calcium channels interact with glycolysis.

To the best of our knowledge, this is the first comprehensive description of *in vitro* effects of LPA on human sperm. LPA significantly improved the motility parameters of sperm, and this effect was closely associated with the regulation of glycolysis and L-type calcium channels in sperm. Our results provide novel insights into the regulatory mechanisms of sperm motility and open up avenues for the development of LAP as a potential therapeutic target to alleviate male infertility problems.

## Data Availability Statement

The original contributions presented in the study are included in the article/supplementary material. Further inquiries can be directed to the corresponding authors.

## Ethics Statement

The studies involving human participants were reviewed and approved by the Ethics Committee on human subjects of International Peace Maternity and Child Health Hospital (GKLW2018-03). The patients/participants provided their written informed consent to participate in this study.

## Author Contributions

YZ, HH, XLZ and YLL conceived and designed the experiment. YLL, LJ and YQL performed the laboratory studies and data collection. JQ, ZW, XGZ and CX participated in data analysis and interpretation. YZ, HH, XLZ and YLL wrote the manuscript. All authors made substantial contributions in critically revising the manuscript and approved the final manuscript. All authors contributed to the article and approved the submitted version.

## Funding

The work was supported by the National Key R&D Program of China (grant no. 2018YFC1005001), the National Natural Science Foundation of China (grant nos. 31871165 and 32071131) and the Shanghai Science and Technology Innovation Action Plan (grant no. 21140903900).

## Conflict of Interest

The authors declare that the research was conducted in the absence of any commercial or financial relationships that could be construed as a potential conflict of interest.

## Publisher’s Note

All claims expressed in this article are solely those of the authors and do not necessarily represent those of their affiliated organizations, or those of the publisher, the editors and the reviewers. Any product that may be evaluated in this article, or claim that may be made by its manufacturer, is not guaranteed or endorsed by the publisher.
